# The Prevalence of Antibodies against Sandfly Fever Viruses and West Nile Virus in Cyprus

**Published:** 2019-03-30

**Authors:** Gaetan Billioud, Christina Tryfonos, Jan Richter

**Affiliations:** Department of Molecular Virology, Cyprus Institute of Neurology and Genetics, Nicosia, Cyprus

**Keywords:** Phleboviruses, West-Nile virus, Cyprus, Seroprevalence

## Abstract

**Background:**

Sandfly fever is an incapacitating disease caused by sandfly-borne Phleboviruses that can lead to meningitis, encephalitis or meningoencephalitis. West Nile virus (WNV), a mosquito-borne Flavivirus, can induce neuroinvasive disease manifested by meningitis, encephalitis or acute flaccid paralysis. Both vectors are endemic in Cyprus and very active during summer. The aims of this study were to determine first the prevalence of sandfly fever viruses (SFV) and WNV infections in Cyprus and second, to investigate their role in central nervous system (CNS) infections.

**Methods:**

For the prevalence study, 327 sera collected in 2013 and 2014 were tested for anti-SFV and anti-WNV IgG using indirect immunofluorescence assay and ELISA, respectively. In order to investigate a possible role of SFV and WNV in CNS infections, 127 sera of patients presenting symptoms of SFV or WNV infections were screened for IgM specific to SFV and WNV.

**Results:**

The overall anti-SFV IgG seroprevalence was 28% and was increasing with age (P< 0.01). The seroprevalence rate for anti-WNV IgG in Cyprus was 5%. Concerning the role of SFVs in CNS infections, anti-SFV IgM was detected in 8 out of 127 sera from selected patients presenting relevant symptoms of infections during vector’s active period. In addition, anti-WNV IgM were detected in 17 out of the 127 patients with compatible symptoms.

**Conclusion:**

The findings confirm the presence of sandfly fever and WNV in Cyprus and should, therefore, be considered in the differential diagnosis of patients with febrile illness/meningitis.

## Introduction

Sandfly (Diptera: Psychodidae)*-*transmitted phleboviruses may cause a transient and moderate febrile illness (named pappatasi fever, 3-day fever or sandfly fever) but can also affect the central nervous system (CNS) leading to serious infections. The most common vectors are *Phlebotomus papatasi*, *P. perfiliewi or P. perniciosus.* The genus *Phlebovirus* has been recently assigned to the newly created family *Phenuiviridae* and the order *Bunyavirales* (1).

Sandfly-borne phleboviruses are enveloped negative-sense and single-stranded tripartite RNA viruses classified around two viral species (Sandfly fever Naples (SFNV) and Salehabad) and two tentative species (Sicilian and Corfu).

Infections with SFNV and Sandfly fever Sicilian (SFSV) viruses are clinically similar and characterized by high fever, headache, retro-orbital pain, malaise, diarrhoea, myalgia, photophobia and anorexia along with thrombocytopenia, leukopenia and elevated liver enzymes (2, 3). Toscana virus, a member of the Naples serogroup, generally induces a mild febrile illness without CNS involvement.

However, when the CNS is affected, high fever, headache, nausea, vomiting, Kernig signs, neck rigidity, myalgia and sometimes unconsciousness, tremors, paresis and nystagmus as well as encephalitis, severe meningoencephalitis and long-lasting sequelae have been reported (4, 5). The Mediterranean basin is endemic for sandfly-borne phleboviruses infections (4, 6–10) and particularly Cyprus where phlebotomines (11, 12) and outbreaks have been reported (13–16).

Regarding West Nile fever, the majority of clinical cases are mild and present with flu-like symptoms characterized by fever, malaise, and myalgia frequently accompanied by rash. Severe cases with signs of encephalitis, meningoencephalitis or meningitis are rare (<1%), most often observed among elderly and associated with a 10% fatality rate (17). The disease results from an infection by West Nile Virus (WNV) (genus *Flavivirus*, family *Flaviviridae*, order unassigned) transmitted through a mosquito (Diptera: Culicidae) bite, mostly from the genus *Culex* and usually from the *Culex pipiens* complex (18), abundant in Cyprus (19, 20). Human infection is solely incidental of the enzootic transmission cycle between avian natural hosts and mosquito vectors (21). The virus is considered to be one of the most important emerging arboviruses in recent years because of the multiple outbreaks reported in Europe and particularly in the Mediterranean area (Greece, Turkey, Spain) (22, 23). To date, no epidemiological data for WNV have been reported for Cyprus but the first neuroinvasive case was reported in 2016 (24, 25) and a second confirmed case in 2018 (ECDC update), confirming the presence of the WNV in the island.

Because of outdated or absent epidemiological data concerning Cyprus, a cross-sectional serologic survey was conducted to estimate SFV and WNV IgG respective seroprevalences in order to assess the exposure of the Cypriot population to these pathogens. In addition, the possible role of these pathogens in CNS infections in Cyprus was investigated by testing for markers of recent infection (IgM) and viral RNA in samples of patients with febrile illness and/or CNS infection.

## Materials and Methods

### Sample collection

**Fig. 1.**
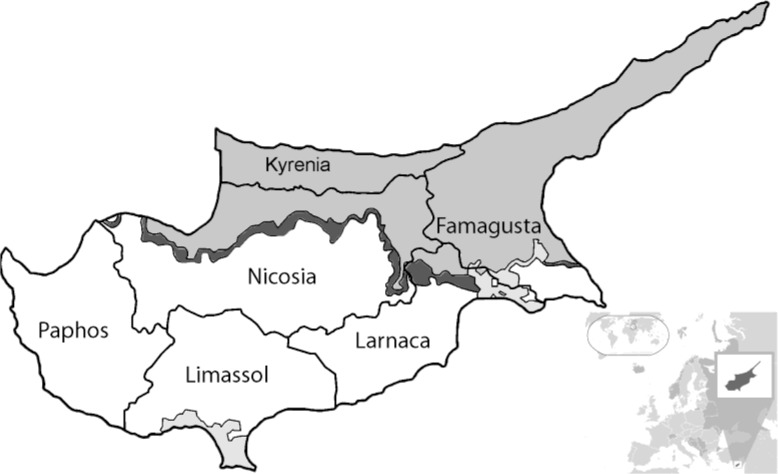
Illustrative map of Cyprus’ districts. White, Area studied; Grey, Non-government controlled area; Dark grey, UN buffer zone; Light grey, UK sovereign bases

Serum and cerebrospinal fluid (CSF) samples from patients were received at the Cyprus Institute of Neurology and Genetics, stored appropriately at −20 °C and used subsequently. For IgG seroprevalence, 327 samples between Jan 2013 and Dec 2014 were retrospectively selected following a leftover sampling methodology matching the 2013 census Cypriot demography according to patient’s sexes (male or female), age and origin of the sample (Famagusta, Larnaca, Limassol, Nicosia or Paphos district) (Fig. 1). Informed consent was taken from the patients before participation.

The patients with known disease, medical procedure or treatments affected the antibodies detection were excluded (i.e. autoimmune disease, immunomodulatory treatments, transplanted, neoplasm, etc.). Determination of specific IgM was performed on samples received during 2013 and 2014 vector active season (warm months, from May to Nov in Cyprus) from 127 selected patients with infectious pathologic features relating to Sandfly or West Nile fevers. Based on similar selection criteria, 72 CSF samples from patients collected between 2015 and 2016 during vector active season were tested for circulating viral RNA.

### SFV IgG and IgM detection

Overall, 327 and 127 sera were analyzed for the presence of anti-SFV IgG and IgM, respectively, by indirect immunofluorescence test (IIFT) BIOCHIP Mosaic™: Sandfly Fever Virus IgG or IgM (EUROIMMUN, Lübeck, Germany). This test is the only commercially available test that offers the possibility to assess the presence of antibody reacting against two species members of the Sandfly Sicilian serogroup (SFSV and Sandfly fever Cyprus viruses, SFCV) and two members of the Sandfly Naples serogroup (SFNV and Toscana virus, TOSV), without excluding the possibilities of cross-reactivity.

Sera were diluted 100 and 50 times in sample buffer for IgG and IgM, respectively, and tested according to the manufacturer’s instructions. Before determining specific anti-SFV IgM, IgG antibodies and rheumatoid factors of class M were removed by immune-absorption using the Siemens RF-Absorbent (Marburg, Germany) [Ref: OUCG194] for 15min at room temperature. Samples were considered positive if in some cells, but not all, cytoplasmic finely granular structures and inclusion bodies fluoresced with the same pattern obtained as for the positive control serum.

### WNV IgG and IgM detection

Overall, 327 sera were collected for specific anti-WNV IgG detection and 127 sera for IgM detection, respectively, using an FDA-approved enzyme-linked immunosorbent assay (ELISA) (EUROIMMUN, Lübeck, Germany). Sera were diluted 100 times for both IgG and IgM in sample buffer (containing IgG/RF-Absorbent for IgM detection) and tested according to the manufacturer’s instructions. The samples were considered positive when the ratio between the extinction values of the samples over the calibrator (20 RU/ml for IgG) was ≥ 1.1.

### Viral RNA extraction and RT–PCR amplification

Viral RNA was extracted from 72 CSF samples using 400μl of clinical specimen by the iPrep™ PureLink® Virus kit according to manufacturer’s instructions and resuspended in 100μl DEPC water. Ten microliters of the extracted viral RNA was used as a template using specific primers with the SuperScript® III Platinium One-Step qRT-PCR kit (Invitrogen, USA), according to the manufacturer’s instructions. For Phlebovirus detection and quantification, the specific primers used amplified the 3′ end of the small segment N gene and were based on published protocols (26, 27). For *Flavivirus* detection and quantification, the specific primers targeted the NS5 gene and were based on a previously published protocol (28).

### Statistics

Results were statistically analyzed with the Prism™ version 5.01 software from GraphPad (La Jolla, CA, USA). Along with descriptive statistics, univariate analysis was conducted on the results obtained from the seroprevalence study by Fisher’s exact tests. Comparison of groups’ average age was analyzed by nonparametric two-tailed Mann-Whitney tests. The logistic regression model was generated from the GNU package R environment. P< 0.05 indicated statistical significance.

## Results

### Prevalence study of anti-sandfly fever viruses and West Nile virus IgG antibodies

To determine the exposure of Cypriots to sandfly fever viruses, 327 human serum samples from 2013–2014 were screened for anti-SFVs and anti-WNV IgG. The sampling was distributed by sex and age proportional to Cyprus’ population (Table 1). By sex, 166 were females (∼51%) and 161 males (∼49%). By age groups, 81 were <20yr old (∼25%), 184 aged 20–60yr (∼56%), and 62 were aged >60yr (∼19%) with an overall mean age of 37yr old. By districts, 9 specimens were from Famagusta (∼3%), 32 from Larnaca (∼10%), 107 from Limassol (∼33%), 170 from Nicosia (∼52%) and 9 were from Paphos District (∼3%) (Fig. 1).

**Table 1.** Sandfly fever virus and West Nile IgG seropositivity

	**Tested**	**SFV IgG**		**WNV IgG**	

		**Positive**	**%**	**Positive**	**%**
**Total**	327	93	∼28	16	∼5
**Males**	161	52	∼32	6	∼4
**Females**	166	41	∼25	10	∼6
**< 20 y**	81	6 (*)	∼7	4	∼5
**20–60 y**	184	60 (*)	∼33	11	∼6
**> 60c y**	62	27 (*)	∼44	1	∼2
**Famagusta**	9	0	0	0	0
**Larnaca**	32	6	∼19	1	∼3
**Limassol**	107	33	∼31	5	∼5
**Nicosia**	170	51	∼30	10	∼6
**Paphos**	9	3	∼33	0	0

SFV, sandfly fever viruses; WNV; (*), Statistically significant (P< 0.05)

The overall seroprevalence of anti-SFVs IgG was 28% (CI95%: 23.5–33.4%) and increased with age (Table 1). Difference of mean age in anti-SFV seropositive group (47yr old) compared to the seronegative patient’s group (33yr old) was statistically significant (P< 0.0001). Logistic regression model confirmed the trend (P< 0.001) and computed an increased risk of ∼3% per year (SE 0.6%). Differences observed in seroprevalence for anti-SFV IgG between females (∼25%) and males (∼31%) or between districts (Famagusta: ∼0%, Larnaca: ∼19%, Limassol: ∼31%, Nicosia: ∼30% and Paphos: ∼33%) were not statistically significant (P> 0.05) (Table 1). Difference in prevalence between sera reacting only against SFSV and/or SFCV (∼12%) and sera reacting only against SFNV and/or TOSV (∼11%) was not statistically significant (P> 0.05) (data not shown).

The same 327 human sera from 2013–2014 were also screened for anti-WNVs IgG. The overall seroprevalence of anti-WNV IgG was ∼5% (CI95%: 2.5–7.2%) (Table 1). The difference of mean age in anti-WNV seropositive group (33yr old) compared to the seronegative patients group (37yr old) was not statistically significant. Differences in frequencies observed between each age group, sex and district were also not statistically significant.

Five patients (∼1.5%) were found to have IgG antibodies reacting against both SFVs and WNV (data not shown), giving seroprevalence rates of ∼31% (5/16) and ∼5% (5/93) of anti-SFVs and anti-WNV IgG in anti-WNV and anti-SFVs IgG positive groups, respectively. The differences of rates in positive groups compared to the general population (∼28% and ∼5% for anti-SFV and anti-WNV IgG, respectively) were not statistically significant.

### Role of SFV and WNV infections in CNS infections in Cyprus

To assess the morbidity of SFV infections in Cyprus, human sera from patients displaying infectious pathologic features in 2013 and 2014 during sandflies’ active period were tested for markers of recent SFV infection, IgM. Eight sera (∼6%) were found positive for anti-SFV IgM out of the 127 selected patients (Table 2). Difference of SFV IgM seroprevalence rates between each age group, sex and district were not statistically significant. However, a higher frequency of samples reacting only against SFV serotypes belonging to the Naples serogroup (SFNV and TOSV, 7/127) than to the Sicilian serogroup (SFSV and SFCV, 0/127) (data not shown) was statistically significant (P< 0.05). Three of the 8 anti-SFV IgM positive patients (∼38%) showed neuroinvasive features (meningitis or meningoencephalitis) but no statistical significance was found compared to the anti-SFV IgM negative group (34/127, ∼26%, P> 0.05) (data not shown). Of the 8 seropositive patients for anti-SFV IgM, four patient’s CSF were available and three of them were also found positive for anti-SFV IgM (data not shown). All CSF positive for SFV IgM reacted only against the Naples serocomplex, further confirming their neuroinvasion capabilities.

**Table 2.** Sandfly fever virus and West Nile IgM seropositivity

	**Tested**	**SFV IgM**		**WNV IgM**	

		**Positive**	**%**	**Positive**	**%**
**Total**	127	8	∼6	17	∼13
**Males**	58	6	∼10	5	∼9
**Females**	69	2	∼3	12	∼17
**< 20 y**	52	2	∼4	8	∼15
**20–60 y**	54	4	∼7	7	∼13
**> 60c y**	21	2	∼10	2	∼10
**Famagusta**	5	0	0	2	∼40
**Larnaca**	10	0	0	0	0
**Limassol**	41	6	∼15	9	∼22
**Nicosia**	66	2	∼3	6	∼9
**Paphos**	5	0	0	0	0

SFV, sandfly fever viruses; WNV

Detection of *Phlebovirus* nucleic acids by specific quantitative RT-PCR in 72 human cerebrospinal fluid samples from patients displaying infectious pathologic features in 2015 and 2016 during sandflies’ active period was performed, but no specimen was found positive (data not shown).

Seventeen (∼13%) out of the 127 tested human sera from patients displaying infectious pathologic features in 2013 and 2014 during mosquito’s active period were found positive for anti-WNV IgM (Table 2). The difference of WNV IgM seroprevalence rates between sexes, age groups or districts were not found statistically significant. Two of the 17 anti-WNV IgM positive patients (∼12%) showed neuroinvasive features (meningitis or meningoencephalitis) but no statistical significance was found compared to the anti-WNV IgM negative group (29/110, ∼26%) (data not shown).

With regard to nucleic acid testing, no WNV RNA was detected in the 72 human CSF samples from selected patients displaying infectious pathologic features in 2015 and 2016 during mosquitos’ active period (data not shown).

## Discussion

Because the presence of arboviruses in Cyprus had been reported previously (15, 24, 29–32), we evaluated the residents’ exposure to SFV and WNV following Cyprus’ demography in terms of age, sex and location.

The overall seroprevalence rate for anti-SFVs IgG estimated in this study was ∼28%. Higher seroprevalence of antibodies against SFSV virus (∼62%) and neutralizing antibodies against SFNV (∼57%), SFSV (∼32%) and TOSV (∼20%) viruses were reported in previous studies on Cypriot residents (33, 34). A recent seroprevalence study of phleboviruses in Cypriot dogs indicated also high neutralizing rates for SFSV (∼60%) and TOSV (8.4%) (35). Lower rates found here may reflect the different specificity and sensitivity of the assay used (IIFT or plaque neutralization reduction assay (PRNT) (36), sampling method skewed towards specific geographical areas (Famagusta, Larnaca or Paphos districts), test subjects (healthy donors or symptomatic patients), species tested (humans or dogs) or simply a lower exposition of the modern Cypriot population, possibly due to arthropods abatement programs to eradicate malaria in the island (37).

Direct comparison with studies depicting SFV seroprevalences from neighbouring countries is limited, mostly because of the wide use of PRNT and the focus on TOSV. However, our study reports comparable anti-SFV IgG seroprevalence rates described in Turkish Central/Northern Anatolia (∼33%) (38) but lower compared to the Turkish Mersin Province (∼67%) (39), both tested by IIF.

The anti-SFV IgG seroprevalence rate increasing with age is in agreement with the conclusions of previous studies (40, 41) and demonstrates that the Cypriot population is exposed to SFVs throughout life. Unfortunately, other risk factors previously identified such as living in rural areas or high levels of outdoor activity could not be assessed because of the lack of patient’s information.

Concerning WNV, the overall IgG sero-prevalence found in Cyprus was 5% and the difference of rates between sexes, age groups or districts were not statistically significant. It is the first time that anti-WNV IgG seroprevalence has been estimated in Cyprus. WNV-specific IgG rates were lower in neighbouring countries such as Greece (2.1%) (42), Northern Italy (0.3–2.1%) (43) or South-Eastern France (1.4%) and higher in Libya (13.1%) or Tunisia (12.5%) (44) but more similar to Turkey (2.4– 12.1%) (20, 45), Algeria (6.7%) or Serbia (∼4%) (46).

In addition, the SFV IgG seropositivity rate in the WNV IgG seropositive group (∼31%) was comparable to the rate found in the entire population (29.4%). Similarly, the WNV IgG seropositivity proportion in the SFV IgG seropositive group (∼5%) was analogous to the sample population tested in this study (∼5%). This supports the hypothesis of random chance to contract each viral infection.

Regarding the role of SFV in CNS infections in Cyprus, we identified samples (8/127) with markers of recent SFV infection (IgM) in patients presenting symptoms akin to sandfly fever, supporting a role of SFVs in the aetiology of febrile illnesses/meningitis in Cyprus.

Distinction between antigenically related viruses amongst the sandfly fever Naples species (i.e. SFNV and TOSV) and virus closely related to Sandfly fever Sicilian virus (i.e. SFSV and SFCV) based on IIFT can be difficult due to cross-reactivity. However, there was a significantly higher rate of sera from symptomatic patients tested for anti-SFV IgM reacting only against viruses from the Naples serogroup than sera reacting only against viruses belonging to the Sicilian serogroup. Because of similar rates observed for IgG between serogroups and that patients were selected based on relevant symptoms, it confirms that infections from viruses belonging to the Naples serogroup are more symptomatic, particularly TOSV (47). Anti-SFV IgM specific to members of the Naples serogroup are at higher concentrations or last longer than the ones specific to Sicilian-like viruses (34). However, the detection of anti-SFV IgM specific to the Naples serocomplex in the CSF of affected patients strongly supports their neuroinvasive pathogenesis.

Possible cross-reactivity with anti-Hantavirus and anti-Crimean-Congo hemorrhagic fever virus (CCHFV) immunoglobulins cannot be ruled out based on the IIFT assay used. However, no hantavirus or CCHFV infection nor hemorrhagic fever have ever been reported in Cyprus so far. Even though their vectors are present on the island (48), it is unlikely that the anti-SFV antibodies seroprevalence rates depicted in this report are markedly influenced due to such cross-reactivity.

Markers of recent WNV infection (IgM) were also detected in patients displaying relevant symptoms, highlighting its circulation and morbidity in the Cypriot population. Few seropositive patients had neurological symptoms such as meningitis or meningoencephalitis. Together with the first Cypriot symptomatic case of a 75yr-old man with confirmed WNV infection during summer 2016 (24), these results demonstrate the circulation of WNV in Cyprus. All the anti-WNV IgM seropositive cases were from 2013, following the trend of marked decreased West Nile fever autochthonous cases reported in Europe during these yr (49).

Attempts to detect viral genetic material in more recent (2015 and 2016) patients’ CSF by RT-qPCR following published protocols (26–28) were unsuccessful. The most likely causes for this shortcoming are probably low SFV and WNV viremia detectable only in a small period of time, usually before patients present to the healthcare personnel, the limited number of samples tested and the use of direct RT-qPCR.

## Conclusion

This report provides evidence of significant SFV and WNV circulation in Cyprus as well as evidence of their pathogenicity among Cypriot patients. For these reasons, the surveillance and study of arboviral infections in Cyprus should be strengthened as these pathogens are potential causes of incapacitating and severe neurological diseases.
